# Increased temporal binding during voluntary motor task under local anesthesia

**DOI:** 10.1038/s41598-023-40591-x

**Published:** 2023-09-04

**Authors:** Karina Kirk Driller, Camille Fradet, Nina Mathijssen, Gerald Kraan, Richard Goossens, Vincent Hayward, Jess Hartcher-O’Brien

**Affiliations:** 1 Faculty of Industrial Design Engineering, Delft University of Technology, 2628 CE Delft, The Netherlands; 2 Institut des Systèmes Intelligents et de Robotique (ISIR), Sorbonne Université, 75005 Paris, France; 3 Reinier Haga Orthopaedic Centre, 2725 NA Zoetermeer, The Netherlands

**Keywords:** Human behaviour, Sensorimotor processing, Sensory processing

## Abstract

Temporal binding refers to a systemic bias in the perceived time interval between two related events, most frequently voluntary motor actions and a subsequent sensory effect. An inevitable component of most instrumental motor actions is tactile feedback. Yet, the role of tactile feedback within this phenomenon remains largely unexplored. Here, we used local anesthesia of the index finger to temporarily inhibit incoming sensory input from the finger itself, while participants performed an interval-estimation task in which they estimated the delay between a voluntary motor action (button press) and a second sensory event (click sound). Results were compared to a control condition with intact sensation. While clear binding was present in both conditions, the effect was significantly enhanced when tactile feedback was temporarily removed via local anesthesia. The results are discussed in light of current debates surrounding the underlying mechanisms and function of this temporal bias.

## Introduction

*Temporal binding* can be described as a perceptual illusion in the temporal domain characterized by a compression of the perceived time interval between two causally related events^1,2^. In the context of voluntary actions and their external sensory outcome, this phenomenon is also widely known as *intentional binding*. It is often considered to be an implicit marker of agency, that is, the conscious experience of bringing about changes in the external world through voluntary actions^3,4^. This link has, however, recently been questioned^5,6^. Temporal binding has been shown to be altered in certain psychiatric disorders, most notably schizophrenia^7,8^, and has been linked to personality traits in non-clinical individuals too^9–11^. Extensive research has been conducted to uncover the factors that influence this phenomenon.

Previous research has highlighted the importance of intentionality and volition, such as free choice of action and outcome pursued as well as motor control over the action and outcome prediction for achieving robust binding^3,12–18^. While voluntary motor actions produce binding, involuntary motor actions (i.e., via TMS of motor cortex) tend to produce reverse binding^3^. Similarly, sensory events exogenous to the agent (e.g., tactile sensations and auditory signals) in the absence of motor movements lead to repulsion rather than a binding effect^12,13^. Furthermore, self-associated stimuli have been shown to produce stronger binding than stimuli associated with others^19^, although there are inconsistencies among the findings^20^. Finally, researchers have emphasized the importance of a causal or contingent relationship between action and outcome for temporal binding^21–25^. Consequently, while intentional movement is necessary, it may not be sufficient to induce binding if the outcome is not perceived to be action contingent. These studies stress the favorability of an overall naturalistic experimental setup since naturalistic events tend to be associated with inherently plausible event links^26^.

An inherent aspect of instrumental, voluntary motor actions such as pressing a button or snapping a twig, is immediate tactile feedback that, alongside proprioceptive signals, contributes to an awareness of having changed the state of the world. To date, only limited research has examined the role of tactile information in temporal binding during a voluntary motor task, and the results have been contentious. While some research has indirectly suggested the importance of tactile sensations in temporal binding^27–30^, only a few studies have attempted to directly manipulate tactile signals. This latter research, which has utilized mid-air, contactless gesture-based tactile interactions, has led to incongruent findings, reporting either no binding for contactless interactions^31^ or no difference between contactless interactions and physical keypress control conditions^32^. Contactless interactions fundamentally differ from mechanical interactions, leading to limited mechanistic insights and potential confounds. Despite the steady development and expanding applications of mid-air contactless interfaces, mechanical button presses constitute a more common interaction condition encountered by most people on a daily basis.

To address the role of tactile feedback in temporal binding, the current study took the approach of directly abolishing local tactile information during a voluntary motor task, while leaving the long-range consequences of the action intact. Using local anesthesia of participants’ index finger, we measured the perceived temporal attraction between an action (button press) and a sensory outcome (click sound), while preserving all other aspects of a mechanical button press. The ‘anesthesia’ condition was compared to an ‘intact’ control condition. Since the muscles that move an index finger are located entirely in the hand and forearm, we could assume that the participants’ motor behavior would be unaffected by the local anesthesia, except for motor control directly linked to afference from the finger itself^33–35^. Because local anesthesia can result in a perceived enlargement of the anesthetized body part^36^, participants also estimated their perceived finger length in the two conditions.

Recent research has cast doubt on the link between temporal binding and agency or intention-related aspects of behavior. This research has emphasized the role of causal inferences and processes of multisensory integration, arguing that substantial evidence ought to be provided when claiming effects to be more than multisensory causal binding^5,6^. In the present study, no preliminary assumptions were made about such a relationship. Following this reasoning we therefore adhere to the term “temporal binding” instead of the more commonly used term “intentional binding” when referring to the mere effect of a perceived compression of the time interval between a voluntary motor action and sensory outcome.

## Methods

### Participants

Fifteen participants were recruited for the study. None of the participants reported psychiatric or neurological disorders, a history of finger/hand/upper limb trauma, or any condition affecting normal sensorimotor function. One participant was excluded due to incomplete local anesthesia, resulting in a final sample of fourteen participants (8 female, 6 male). The mean age of this remaining cohort was 26.72 (SD 9.24). As assessed by the Edinburgh Handedness Inventory^37^, one participant was left-handed, one participant was mixed-handed, and the remaining twelve participants were right-handed. The mixed-handed participant reported being right-handed and therefore performed the experiment with the right index finger.

The study was approved by the Dutch Medical Ethics Review Committee METC-LDD. Data were collected at the Reinier Haga Orthopaedic Centre and the study was approved by the local ethics committee. The study is also registered on the website of ClinicalTrials.gov with the identifier NCT05253508 (date of first registration 23/02/2022). The methods of this study were performed in accordance with the relevant guidelines and regulations and in accordance with the Declaration of Helsinki. All participants gave written informed consent prior to the start of the experiment and preparation.

### Apparatus

**Figure 1:**
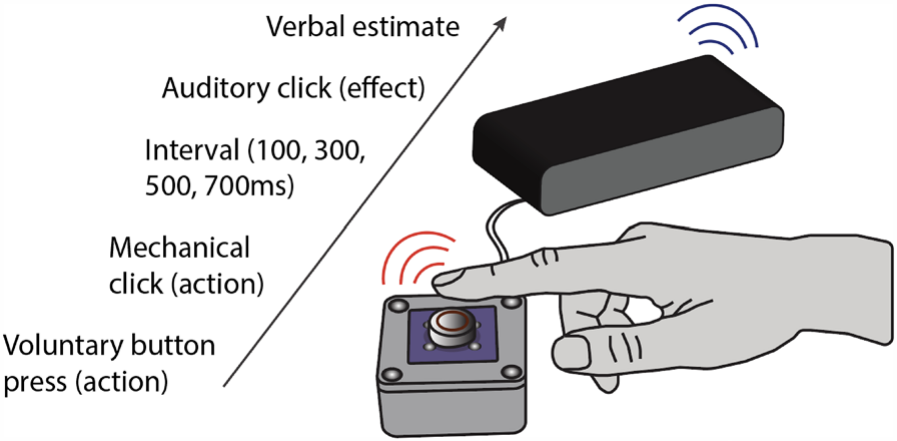
Setup and trial structure. Upon pressing the button, an instant mechanical “click” (action) with audible and tactile detent was emitted and then followed by a delayed auditory “click” sound (effect) either 100, 300, 500, or 700 ms after the action. Participants then estimated the interval between their own action and the effect in milliseconds.

Participants were seated in front of a computer screen, and a snap action switch button was placed in front of their dominant hand, as shown in Fig. 1. When pressed and released, the button responded with a sharp audible click and a clear tactile detent. The switch button was connected to a digital signal processor (Bela, Augmented Instruments Ltd, Mile End Road, London, England) that sensed its state at a rate of 44,100 samples per second. Upon release, a second auditory click was produced through a loudspeaker with delays of 100, 300, 500, or 700 ms. A Matlab^®^ (MathWorks, Natick, Massachusetts, USA, Version R2021b) application, communicating with the digital signal processor through a User Datagram Protocol (UDP), specified the delay between the button press and the auditory click, and collected the responses. Once this parameter was transmitted, the trial started and only the digital signal processor was involved, whose inner latency is systematically less than 1 ms. Participants were able to see their hands throughout the experiment.

### Preparation

Local anesthesia was induced via a two-sided subcutaneous digital nerve block resulting from an injection of 1–2 ml of ropivacaïne 0.2% or lidocaine 2% at either side of the palmar root of the dominant index finger^38^. The effect of the digital nerve-block was assessed using Semmes–Weinstein monofilaments examination. Anesthesia was considered complete when no sensation was reported to remain in the second and third phalanx. One participant had to be excluded because a complete digital nerve block could not be achieved. A complete digital nerve-block was achieved for the remaining fourteen participants. On one of these fourteen participants, the nerve block was done with two injections of lidocaine only. A second participant received a second dose of lidocaine (4 injections in total), when showing early recovery during the data collection of the preceding experiment. The remaining twelve participants received two injections of ropivacaïne each. For seven of these participants, sensation was completely abolished on three phalanges. This group reported a mild effect of the anesthetic on the adjacent side of the middle finger. For the remaining seven participants, some sensation was reported on the dorsal side of the proximal phalanx, close to the knuckle. This variation is considered normal due to the cutaneous innervation of this specific region. Small, dorsal branches of the radial nerve may depart from primary branches located proximal to the injection site and will thus eventually not be blocked by the palmar approach of the digital nerve-block^39,40^. No distinction between these groups was made in the analysis.

### Experimental design and procedure

We used a verbal interval estimation paradigm which has been validated in prior studies on temporal binding^41–44^. At the beginning of each trial, a green window appeared on the screen placed in front of participants to indicate that testing could start. Participants then pressed the button to initiate a trial whenever they liked. The button responded with an immediate audible and palpable action-click when successfully pressed, which was necessary as an objective marker for the start of the interval for both conditions. A second effect-click sound was then emitted with a delay of 100, 300, 500, or 700 ms. Participants’ task was to estimate the interval between the button press and the resulting effect-click in milliseconds. They were asked to provide estimates that were as accurate as possible. Estimates were provided verbally and recorded by the experimenter. The setup and trial structure are illustrated in Fig. 1.

Participants were told that the time interval between the button press and the effect-click could be any random value between 10 and 1000 ms. Prior to testing, all participants were given examples of real time intervals of 10, 500, and 1000 ms. To familiarize themselves with the delay space, they pressed the button and heard the response for each of these intervals as many times as they felt it necessary.

The experiment adopted a repeated-measures design in which all participants took part in two conditions, ‘anesthetized’ and ‘intact’. The order of these conditions was balanced across participants. The experiment comprised two separate blocks of sixty trials encompassing all delays. Each delay was repeated fifteen times in a randomized order. The blocks were administered on different days, separated by one to three days. This minimum 24-h period was maintained because the effect of ropivacaine can last up to 23 h. Due to practical constraints, two participants completed only forty trials per block. Prior to testing, the participants’ index finger length was measured. Then the temperature of their index finger pad was recorded with an infrared thermometer (Tacklife IT-T09) and its hydration level was measured with a skin hydration measurement instrument (Corneometer^®^ CM 825). Participants also assessed the perceived length of their index finger by adjusting the arm of a caliper with both their hands and the display concealed. Temperature, hydration level, and perceived finger length were recorded three times per condition and per participant.

The present experiment was conducted after a material-property discrimination experiment reported elsewhere and approximately 1 ½ hours after the application of the digital nerve block. This other experiment involved exploring surfaces with the index finger, meaning that all participants had the chance to become familiar with the novel experience of an anesthetized finger ahead of the data-collection. The Semmes–Weinstein monofilament examination and all finger-pad temperature and moisture measurements reported here were taken just prior to data collection of the present experiment.

### Statistical analyses

Differences in finger temperature, hydration level, and perceived finger length between the two conditions (anesthesia/intact) were assessed using paired-sample *t*-tests. A linear-trend analysis with an exclusion criterion of R^2^ > 0.5 was carried out for each participant and condition to ensure that there was a significant trend of gradual increase in the estimates of the 100, 300, 500, and 700 ms intervals. Furthermore, interval estimates that were three or more standard deviations from the mean were excluded. The remaining estimates were subjected to an ANOVA with repeated measures on factors *condition* (anesthesia/intact) and *delay* (100, 300, 500, 700 ms). A second ANOVA with repeated measures on *order* (first block/second block) and *delay* (100, 300, 500, 700 ms) was performed to control for any order effects, although the condition *order* was balanced. The significance level was set at p < 0.05. Sphericity was assessed using Mauchly’s test, and adjustments were made using Greenhouse–Geisser corrections. Post-hoc Bonferroni-corrected pairwise comparisons were used to determine which conditions differ from one another for significant effects of factors with more than two levels.

All statistical analyses were performed in R (version 4.2.0, R Core Team, 2021).

## Analysis and results

The mean duration of trials from the button release to response logging was 6.33 s (SD 1.46) for the intact condition and 6.69 s (SD 1.08) for the anesthesia condition. The duration of the entire experiment was approximately six minutes for each condition recorded on separate days.

Mean hydration values were 65.96 (SD 28.09) for the intact condition and 25.28 (SD 7.30) for the anesthesia condition. Mean finger pad temperatures were 28.63 (SD 3.10) for the intact condition and 29.81 (SD 2.90) for the anesthesia condition. Paired-samples *t*-tests revealed a significant difference between the hydration measurements (*t*(13) = 5.96, *p* < 0.01) but not the temperature measurements (*t*(13) = − 1.51, *p* = 0.15) for the two conditions. On average, the index finger length was estimated to be 3.18 mm longer than the actual finger length for the intact condition (SD 14.31) and 3.20 mm longer for the anesthesia condition (SD 11.63). A paired-sample* t*-test revealed no significant difference between the two conditions (*t*(13) < 0.01, p = 1.00).

To ensure that there was a significant trend of gradual increase in the estimates of the 100, 300, 500, and 700 ms intervals, a linear trend analysis was performed for each participant and for each condition. No participant had to be excluded based on the criterion of R^2^ > 0.5. Next, interval estimates that differed by more than three standard deviations from the mean were excluded. A total of four responses were excluded on the basis of this criterion. A fifth response was excluded from the analysis due to an undesired disturbance during the data collection.

**Figure 2:**
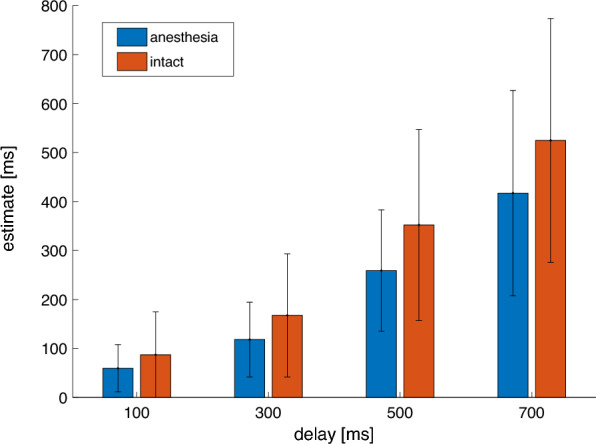
Mean estimated intervals per delay for the anesthesia (blue) and intact (orange) condition. Error bars indicate the standard deviation of the mean.

The remaining interval estimates were subjected to a two-way repeated-measures Analysis of Variance (ANOVA) with factors *condition* (anesthesia/intact) and *delay* (100, 300, 500, 700 ms). The ANOVA yielded a main effect of condition (*F*(1,13) = 5.37, *p* = 0.04, η^2^ = 0.05), indicating shorter interval estimates in the anesthesia condition (mean 213.41, SD 95.14) than in the intact condition (mean 282.96 SD 147.51). The analysis revealed a significant main effect of delay, indicating that longer delays were estimated as longer. This effect did not pass Mauchly’s test for sphericity but remained significant after Greenhouse–Geisser corrections (*F*(1,14) = 49.20, *p*[*GG*] < 0.01), η^2^ = 0.52). For the anesthesia condition, the mean estimates were 59.53 (SD 48.19) at 100 ms, 118.15 (SD 76.26) at 300 ms, 259.14 (SD 123.99) at 500 ms, and 417.22 (SD 209.53) at 700 ms. For the intact condition, the mean estimates were 87.05 (SD 87.51) at 100 ms, 167.62 (SD 125.79) at 300 ms, 352.22 (SD 195.13) at 500 ms, and 524.79 (SD 249.07) at 700 ms. Bonferroni-corrected post-hoc pairwise comparisons for the main effect of delay revealed significant differences between all levels of delay (all p < 0.01). However, the interaction between *condition* and *delay* was not significant (*F*(1,20) = 2.16, *p*[*GG*] = 0.15, η^2^ = 0.01), indicating that the difference in binding between the two conditions was not specific to any delay. A second ANOVA with the factors *order* (first block/second block) and *delays* (100, 300, 500, 700 ms) was carried out to control for any order effects, although the order of the conditions was balanced. The analysis revealed no significant main effect of order (*F*(1,13) = 0.13, *p* = 0.72, η^2^ < 0.01). Figure 2 shows the mean estimated intervals for each condition and delay.

## Discussion

The analysis revealed a significantly *increased* effect of temporal binding in the anesthesia condition as compared to the intact condition. This difference was not specific to any of the delays. Possible mechanisms and implications of these results are discussed below.

Some studies have indirectly suggested the importance of tactile sensory feedback in temporal binding. Increased temporal binding is for instance observed when the voluntary action involves touching skin rather than a button^27,29^. Furthermore, a temporal compression effect can be observed for tactile stimuli delivered to the hand during hand movements^30^. However, few studies have directly manipulated this variable in a temporal binding paradigm. One study used a laser-beam paradigm in which participants could cause a visual stimulus to instantly disappear via mid-air button-press gestures, which then led to the appearance of a second visual target stimulus after a specified delay^31^. In contrast to a control condition which involved pressing a physical key, the contactless key-press gesture did not result in a significant binding effect, leading the authors to conclude that “both intentionality and tactile sensory feedback are necessary to induce the binding effect”. However, another study investigating temporal binding using contactless key-press gestures, reported no statistical difference between this condition and a control condition involving a physical key press action—provided that the outcome stimulus was auditory or haptic in nature^32^. When the actions were followed by a visual outcome, the binding was diminished for either of these conditions. The lack of efficiency of visual targets in producing binding as compared to auditory targets has been reported previously^45,46^, although visual targets have successfully been used elsewhere to induce temporal binding^42,47^. Further differences between the two studies include that Cornelio Martinez et al.^32^ did not provide any initial, action-related, feedback to indicate that the mid-air button-press gesture had successfully been carried out, which may have introduced some inaccuracy for the participants to judge the exact onset of the intervals and higher temporal uncertainty in their motor production estimation^48^.

While we cannot assert what caused these incongruent results, ‘contactless’ gesture-based actions are not directly comparable to mechanical keypress actions. Observers come with a prediction of the consequences of their own actions based on the physics of their everyday environment. In contactless gesture interactions, observers may not apply the same physics and may anticipate different consequences of their actions. In the present study we believe to have mitigated this shortcoming by introducing an endogenous modification in the participant, resulting in sensationless rather than contactless interactions. Here, we did not only observe a significant binding-effect for both experimental conditions, but an increased effect after temporary deafferentation via local anesthesia. While this finding may seem counter-intuitive, considering previous research highlighting the significance of tactile feedback for temporal binding, it is important to bear the differences between those previous studies and the present study in mind. These previous studies either added additional tactile stimulation to a normal somatosensory condition^27,29,30^ or avoided tactile feedback by eliminating physical contact altogether^31,32^. Adding tactile stimulations or feedback in some of the previous studies may for instance have increased the perceived contingency or simply relevance of the interaction in question, leading to a comparatively stronger effect of temporal compression. Similarly, preventing contact entirely may have strained participants sense of control over the event, affected its perceived plausibility, or challenged the inherent cause-effect link between the action and outcome. However, it is unlikely that endogenously suppressing tactile feedback in the present study would have weakened such links. Furthermore, we have no reason to believe that participants’ experience of control or ‘ownership’ over their anesthetized finger was impaired. We readily observed that participants were able to move their anesthetized finger freely, with the exception of mild restrictions due to the swelling of the finger from the anesthetic solution injected at the base. This swelling was however greatest immediately after the injection of the anesthetic and mainly affected full flexion of the finger, which was not required during the task. Moreover, while disproving that tactile sensory feedback is necessary to induce a temporal binding effect, this finding still provides evidence that tactile feedback is functional in determining temporal binding, since diminishing it significantly alters the outcome.

The finding that tactile sensory feedback is not a prerequisite for a stable temporal-binding effect to occur, is furthermore consistent with observations reported in studies of body ownership. Body ownership refers to the experience of one’s body and body parts belonging to oneself^49^, and the phenomenon has frequently, but not always, been argued to be related to agency and temporal binding^50–52^. Whilst it is known that cutaneous stimulation can induce a sense of ownership of an artificial limb in the so-called rubber-hand illusion^53–56^, two studies have shown that tactile sensations do not seem to be essential for an ownership illusion to take place, provided that proprioceptive cues from muscle receptors are present^57,58^. By exciting muscle receptors during movement, the researchers showed how ownership over a plastic finger could reliably be induced, while cutaneous and joint receptors of participants real finger had been blocked using local anesthesia. The present results show how a similar non-conditional relationship seems to exist between tactile sensations and temporal binding. However, while these arguments support our finding that tactile sensory feedback is not strictly necessary for temporal binding to occur, they do not address potential reasons for why the effect is exaggerated under local anesthesia. In the following section, we discuss potential mechanisms that may have contributed to such an effect.

Human body representations are malleable^36,54,57,60^, and perceptual changes associated with local anesthesia have been described previously, some of which may be worth considering here. For example, perceptual distortions of the body image are a commonly reported consequence of local anesthesia. Most notably, it is known that local anesthesia can lead to an increase in the perceived size and shape of the anesthetized body part, as well as changes in its perceived posture^36,59–63^. Gandevia and Phegan^36^, for instance, used a drawing task as well as the selecting of drawings of thumbs to demonstrate that perceived thumb size increases by 60–70% under local anesthesia (using a digital nerve block as in the present study).

In the present study we did not find an effect of the digital nerve block on perceived finger length. However, a study performed by Walsh et al.^63^, in which the perceived enlargement of a finger during a digital nerve block was compared to a saline injection control, reported a significant perceptual enlargement of the finger width but not length, suggesting nonuniform changes in the perceived size of the finger following digital anesthesia.

The exact causes and mechanisms behind these perceptual changes are still unclear, although perceptual enlargements may be a consequence of acute changes in cortical representations^36,63–65^. Walsh et al.^63^ further proposed that when sensory information is lost during local anesthesia, the brain might infer injury and increase the body’s perceptual perimeter to protect it from further injury, since body parts that feel larger may be kept further away from hazardous objects. This is in line with findings showing that partial anesthesia^63^, as well as an elevation of peripheral input through painful cooling and innocuous stimulation of the digital nerve^36^, also result in a perceptual enlargement of the affected digit, albeit smaller than the effect observed under local anesthesia.

While perceived *spatial* distortions resulting from acute deafferentation (e.g., local anesthesia) have been reported regularly, no research yet, to the best of our knowledge, has provided insights into perceived *temporal* distortions resulting from voluntary actions with a temporarily deafferented body part. Our results provide initial insights into such a phenomenon. It is known from research on temporal recalibration that our sensory system is able to rapidly adapt to small inter-sensory asynchronies in order to maintain coherence during multisensory interactions^66–69^. Moreover, recent mechanistic approaches to temporal binding itself have highlighted the role of multisensory cue integration and cross-modal grouping as the driving mechanism behind the phenomenon^2,6,28,70^. A redundant cue from a different modality (here auditory) provided at *both* action and outcome in a delay detection task, can for instance reduce both the perceptual grouping of the action and outcome event as well as explicit ratings of agency over the outcome event^71^. This occurs because the additional cue, rather than the outcome event, is integrated with the action event. While this does not occur when the additional auditory cue coincides with only one of the events (action or outcome), it is still plausible that the lack of tactile feedback during the action in the anesthesia condition of the present study somehow facilitated the linking of action and outcome, resulting in an even stronger binding than in the intact condition. Furthermore, a recent study demonstrated how *lighter* key presses lead to *stronger* action binding (that is, the degree to which the perceived time of an action is shifted forward in the so-called Libet-clock paradigm), when followed by an auditory cue^28^. Such results are in line with the findings reported here, suggesting that somatosensory or tactile feedback is negatively correlated with temporal binding. Whether the increased binding reported by Cao et al.^28^ or in the present study coincided with an exaggerated sense of agency, remains to be tested but could be expected if one adopts a view of the sense of agency as being the general product of causality determination between action and outcome^22,71,72^.

It could finally be reasoned, that increased temporal binding when interacting with objects in the environment with a sensorily affected body part plays a functional role in avoiding perceptual conflicts or in preventing injury. An exaggerated temporal link between one’s actions and the outcome could consequently be useful in confirming that an action has been performed successfully with the deafferented or injured body part, in the absence of tactile feedback. In this view, increased temporal binding during local anesthesia could be seen as a compensatory mechanism to ensure that significant events are linked together when confirmation from one sensory modality is either specifically missing or more generally modulated. If the latter hypothesis holds true, then other somatosensory modulations (such as for example increased tactile stimulation or pain) might also lead to a change in temporal binding. In fact, Faivre et al.^73^ demonstrated how sensorimotor conflicts induced by asynchronous (as opposed to synchronous) tactile stimulation of the trunk or hand while tapping with the finger, led to an increase in temporal binding similar to that observed under local anesthesia. Together with the present results, this may point to a more general account in which the temporal-binding effect is sensitive to sensory modulations. Findings showing that arousal itself can enhance temporal binding^74^ would be in line with such a hypothesis. However, further research would be needed to verify this hypothesis. Research on pain and temporal binding could for instance shed further light on this question. While no research has yet been reported investigating the effect of painful sensations on temporal binding, effects of increased analgesia through an increased sense of agency or control is a well-known phenomenon^75–78^.

It remains to be tested whether the temporal perceptual distortions described in the current study are a direct consequence of the temporary deafferentation (e.g., a protective mechanism) or a more indirect effect, such as a consequence of the better-documented spatial distortions (perceived enlargement of the finger leads to increased temporal binding). Vignemont et al.^79^ demonstrated how spatial distances are perceived to be larger when touched with a body part that is perceived to be larger. In other words, if we believe to have caused an event further away or closer to our body, spatial separation may alter our perceived timing of this event too. In the present study, we must furthermore bear in mind that participants had the opportunity to become familiar with the sensation and the use of their anesthetized finger during a preceding experiment. This prompts the question of the phenomenon’s sensitivity to learning and time. While the effect observed here could be an acute and immediate response to the temporary deafferentation itself, it might also be an effect ascribable to learning and adapting to the new biomechanical constraints and changes in feedback from the anesthetized finger. Future studies will need to investigate the time course of such perceptual changes. Research on temporal binding using tools or prosthetic limbs, as well as research including individuals who have lived with deafferented limbs over a longer period of time, could potentially shed light on some of these questions.

### Clinical considerations

Temporal binding has been reported to be altered in certain psychiatric disorders. Most clinical research has focused on schizophrenia, which has been associated with increased temporal binding^80–82^. However, other clinical populations, such as individuals with apraxia and alien limb^83,84^, Parkinson’s disease^85,86^, autism spectrum disorder^88,89^, Gilles de la Tourette^90^, and individuals with borderline personality disorder traits^91^ and obsessive–compulsive tendencies^87^ have been associated with altered temporal binding effects. Altered temporal binding effects in clinical populations have most frequently been viewed upon in the light of an altered sense of agency. However, our findings once again highlight the importance of not taking this link for given^5,6,21,22,25^. Temporal binding appears to be sensitive to a multitude of factors that may not be directly linked to agency-related modulations. Here, we have shown that it is sensitive to a specific type of sensory alterations. Although we can only speculate, alterations in sensory processing, which is a commonly described symptom in many of the above-mentioned conditions, could for instance be one of several mediating factors in the altered temporal-binding effects observed in certain clinical populations. It would be important for future research to try to uncover such mediating factors where they exist.

### Limitations of the current study and future directions

The present study comprises several limitations. While the interval estimation procedure used in this study has the advantage of being less visually demanding and less sensitive to precise instructions and certain changes in the setup as compared to the Libet Clock paradigm^92,93^, a clear drawback of the interval-estimation procedure is, that it does not allow distinguishing between so-called action-binding from effect-binding. Consequently, we cannot know whether the effect observed in the present study was due to participants experiencing their action as occurring later, the effect as occurring earlier, or a combination of both. Another limitation is, that we did not measure changes in the perceived finger-width, only length, as this would have required a saline control in the intact condition. However, knowledge of this might have facilitated a potential linking of well-known spatial distortions during local anesthesia with the temporal distortions observed here. Finally, it is important to bear in mind that the removal of tactile cues using local anesthesia in the current study did not eliminate all haptic cues. Proprioceptive cues in the hand and forearm, as well as vibratory cues propagating to remote sites, may still have been available^94–97^. Recording of such propagating cues at remote intact sites (such as the back of the hand) could have provided a way to assess their potential role in the observed effect.

## Conclusion

The present study revealed an enhanced effect of temporal binding for motor actions carried out with an anesthetized finger as compared to an intact finger. While this supports the conclusion that tactile sensory feedback is not a prerequisite for temporal binding to occur, it also emphasizes the importance of this parameter in modulating temporal binding. The precise mechanism by which the effect is enhanced when tactile feedback is temporarily removed, remains to be understood. Whether the effect observed in the current study was a mere effect of changes in multisensory causal binding or was in any way linked to changes in action-intentionality or agency (increased sense of “I did that”), remains to be tested. Similarly, future research will need to investigate whether the temporal distortions observed here are related to previously reported spatial distortions under regional anesthesia, or whether these are two independent effects.

## Data Availability

The datasets analyzed are available from the corresponding author on reasonable request.
